# Maternal stress and sex ratio at birth in Sweden over two and a half centuries: a retest of the Trivers–Willard hypothesis

**DOI:** 10.1093/humrep/deab158

**Published:** 2021-07-26

**Authors:** Hanbo Wu

**Affiliations:** Nuffield College and Department of Sociology, University of Oxford, Oxford, UK

**Keywords:** sex ratio, maternal stress, Trivers–Willard hypothesis, Sweden, time series analysis

## Abstract

**STUDY QUESTION:**

Is there a negative relationship, as predicted in the Trivers–Willard hypothesis (TWH), between the intensity of maternal stress and sex ratio at birth (SRB)?

**SUMMARY ANSWER:**

Using a comprehensive data set with multiple indicators of maternal stress, most measures of stress show no statistically significant association with SRB over a period spanning 243 years, indicating no support for the TWH.

**WHAT IS KNOWN ALREADY:**

Evolutionary biologists have proposed a widely discussed hypothesis that women in poor and stressful conditions during pregnancy are more likely to give birth to girls, and exposure to stressful events may therefore lead to a reduction in sex (male-to-female) ratio at birth. The empirical evidence so far is mixed.

**STUDY DESIGN, SIZE, DURATION:**

Annual time series data, spanning 243 years between 1749 and 1991 for Sweden at the national level, were drawn from multiple sources. The outcome is defined as the percentage of male births relative to all births in Sweden in a given year. The covariates include a set of economic and climatic variables as proxies for maternal stress.

**PARTICIPANTS/MATERIALS, SETTING, METHODS:**

We conduct a series of ARIMA (autoregressive integrated moving average) models to examine the relationship between maternal stress and SRB during three periods: 1749–1991, 1749–1861 and 1862–1991.

**MAIN RESULTS AND THE ROLE OF CHANCE:**

In 1749–1991, economic proxies for maternal stress showed no statistically significant association with SRB. In 1749–1861, two indicators were significantly associated with SRB, but the coefficients were opposite in direction to the TWH. In 1862–1991, five out of six covariates showed no significant association with SRB. An additional analysis found no significant correlation between sex ratio of stillbirths and all covariates in 1862–1991. Our results are incompatible with the TWH and suggest that previous findings in support of the TWH are not robust.

**LIMITATIONS, REASONS FOR CAUTION:**

This study provides population-level evidence that may not necessarily reflect the nature of all individuals due to the ecological fallacy. The time series analysed in this study are annual data, and we cannot examine the potential seasonality due to the lack of disaggregated monthly data. Our findings may not be generalised to the contexts of extreme maternal stress conditions such as famine and war.

**WIDER IMPLICATIONS OF THE FINDINGS:**

The results from existing studies in this topic may be speculative, and additional research with more comprehensive design, data and covariates is needed to reconsider the robustness of previous findings.

**STUDY FUNDING/COMPETING INTEREST(S):**

The author receives no external funding and has no conflict of interest to declare.

**TRIAL REGISTRATION NUMBER:**

N/A.

## Introduction

In most human populations, the sex ratio at birth (SRB), also known as the secondary sex ratio, shows both ‘striking stability across time’, hovering around 105 male births per 100 female births (i.e. 51.2% of total births are boys), and ‘significant variation with a number of variables’ (James, 2012). Yet, scholars have made several hypotheses on the causes of short-term variations and have suggested dozens of biological, environmental, demographic, socioeconomic and psychological factors that can affect SRB (James, 1987, 2012; Chahnazarian, 1988; James and Grech, 2017). Generally, these factors produce their effects on SRB either through the alteration of sex ratio at conception or through the modification of sex-selective loss in utero, or both (Chahnazarian, 1988; James, 2012).

A particularly intriguing and influential hypothesis of SRB variations was proposed by Trivers and Willard (1973), suggesting that ‘natural selection should favour parental ability to adjust the sex ratio of offspring produced according to parental ability to invest’, and female mammals including humans are therefore able to adjust offspring sex ratio in response to their maternal condition in order to achieve optimal reproductive payoff. The assumptions made in the Trivers–Willard hypothesis (hereafter the TWH) are that (i) reproductive success of male offspring is more unstable and resource-sensitive than that of female offspring, (ii) maternal condition is associated with the offspring’s condition and (iii) maternal condition is positively associated with reproductive success. Therefore, parental investment in sons will yield greater reproductive payoff with good maternal conditions but lower reproductive payoff with poor maternal conditions, relative to a comparable investment in daughters. As a result, the TWH proposes that mothers are more likely to give birth to sons when they are in good condition during gestation and to daughters when they are in poor condition. At the macro or population level, the TWH predicts a temporary decline in SRB in places where women are subject to unfavourable situations that can be characterised as poor conditions and lead to prenatal exposure to physical and mental stress. The main mechanism for the TWH is often thought to be the maternal-stress-induced prenatal deaths which disproportionately affect male foetuses (James and Grech, 2017). Accordingly, the TWH also predicts an increase in the percentage of male foetal loss during a stressful period.

The TWH has been tested by biologists, demographers, ecologists, economists, epidemiologists, sociologists and statisticians using data from different sources and with various research designs and methods (Song, 2015). Considerable literature has related declines in SRB to idiosyncratic shocks, including warfare and armed conflicts (Ansari-Lari and Saadat, 2002; Zorn *et al.*, 2002; Kemkes, 2006; Valente, 2015), political upheavals (Catalano, 2003), severe famines (Song, 2012), terrorist attacks (Catalano et al., 2005b, 2006) and earthquakes (Fukuda *et al.*, 1998; Torche and Kleinhaus, 2012; Catalano *et al.*, 2013), among others. Not all existing research, however, provides supporting evidence for the hypothesis. A number of studies have questioned the previous findings of SRB declines during times of wars (Graffelman and Hoekstra, 2000; Polasek *et al.*, 2005; Polasek, 2006), political upheavals (Schnettler and Klüsener, 2014) and famines (Zhao *et al.*, 2013). These equivocal results indicate that the relationship between SRB changes and idiosyncratic shocks is sensitive to data and the context under study (James, 2009).

Another line of research highlights the effects of a more common and robust type of maternal stress, economic contraction, on SRB. Based on the TWH, observed SRB in a population is presumed to decline when the population undergoes a contracting economy in which households consume fewer goods and services than needed or expected, which may sufficiently stress women and hence lower SRB. In a seminal work, Catalano and Bruckner (2005) employed a time series model to 129 years of demographic and macroeconomic data from Sweden and reported a positive relationship between percentage change in private consumption and SRB in 1862–1991. In other words, Swedish SRB was higher when the economy expanded and lower when the economy contracted, which is in line with the TWH. Several time series analyses for other countries have been conducted following their work, but the results are rather mixed. Helle *et al.* (2009) found a non-significant relationship between SRB and percentage change in real gross domestic product (GDP) in Finland in 1865–2003. Another study showed no relationship between consumption and SRB in Poland when using annual data for the period 1956–2005 (Żądzińska *et al.*, 2007), while a later investigation conducted by the same researchers using quarterly data for the period 1995–2007 reported a reduction in SRB in Poland four quarters after the occurrence of economic decline (Żądzińska *et al.*, 2011). An integrative review article suggested that the association between economic stress and SRB presented in former research remained largely speculative and acknowledged the needs for more research on this topic (Margerison Zilko, 2010).

In addition to economic stress, another typical but less examined maternal stress is climatic shock. A handful of studies have discovered a relationship between temperature and SRB (Helle *et al.*, 2007, 2009; Catalano *et al.*, 2008; Fukuda *et al.*, 2014), while others have not (Dixson *et al.*, 2011, 2013a,b). Most of these studies have usually relied on limited measures of temperature recorded in just a few locations as a proxy for climatic shock to the entire population. For example, Catalano *et al.* (2008) used annual average temperature in the Tornedalen region of Sweden and Stockholm to represent climatic shock to populations of four Nordic countries. Such proxies may be problematic as they cannot reflect the average situation relating to climate and temperature in a geographically sparse area. In this study, we use a series of refined temperature records.

A few studies have also examined the relationship between maternal stress and foetal death sex ratio. Bruckner *et al.* (2010) reported an increase in the percentage of male foetal deaths in the United States in September 2001 when 9/11 induced widespread feeling of distress among American women. Using monthly data from the state of California, Catalano *et al.* (2005a) found that the ratio of male to female foetal deaths increased during months in which the unemployment rate also increased. The existing research has only looked over a relatively short period because historical data on prenatal deaths are not recorded in many places of the world.

The objective of this study is to retest the TWH that maternal stress leads to decline in SRB with a comprehensive data set that covers a longer period than previous studies and includes a range of indicators reflecting different aspects of maternal stress. Our data set combines demographic, socioeconomic and meteorological time series for nearly two and a half centuries for Sweden covering the period from 1749 to 1991. We conduct time series analyses to explore the relationship between Swedish SRB and a set of proxies for maternal stress in the period 1749–1991 as well as in two sub-periods 1749–1861 and 1862–1991. We also examine the relationship between Swedish sex ratio of stillbirths and maternal stress in 1862–1991 as an underlying mechanism for the TWH. To the best of our knowledge, this study provides, hitherto, the most comprehensive macro-level analysis of national SRB trends in terms of the duration of the period and the number of covariates under investigation.

## Materials and methods

### Study setting

Sweden provides an ideal setting for testing the relationship between economic and climatic stress and SRB for two main reasons. First, the country ‘has enjoyed a relatively high level of political and social, if not economic, stability’ (Catalano and Bruckner, 2005), for which we can focus on the impacts of economic and climatic stress and minimise the consequences of idiosyncratic shocks. Moreover, Swedish population registers provide sex-specific demographic data for a very long period that can date back to the mid-18th century, allowing us to examine SRB trends in a pre-industrial period. The data quality of Swedish population registers is also thought to be ‘satisfactorily good’ during pre-industrial time and almost 100% complete and accurate after 1860 (Sundbärg, 1907; Fellman and Eriksson, 2011).

### Outcome variables

The main outcome variable is annual SRB for the whole Swedish population. Instead of using the conventional expression for sex ratio as the male-to-female ratio, we calculated SRB as the percentage of male births in a given year, which is recommended by methodological literature on statistical analysis of SRB to avoid confusion in interpretation of the results and to facilitate comparisons between studies (Wilson and Hardy, 2002). Annual number of live births by sex was drawn from the Human Mortality Database (HMD, University of California, Berkeley and Max Planck Institute for Demographic Research, 2018), from which we calculated annual SRB as the percentage of male births between 1749 and 1991. The HMD compiles detailed demographic data such as age- and sex-specific births, deaths and population counts from Swedish population registers.

Annual number of stillbirths by sex between 1861 and 1991 was provided by Statistics Sweden, which we used as a proxy for foetal loss. Stillbirths can represent only part of the total prenatal deaths, but this is the most suitable and comprehensive variable we can rely on to examine the foetal loss mechanisms underlying the TWH. In line with the expression for SRB, we calculated the sex ratio of stillbirths as the percentage of male stillbirths in a given year.

### Economic covariates

Eight economic covariates were used in this study to measure the intensity of maternal stress attributable to economic conditions in a given year. First, we used GDP to measure the values of all goods and services produced as an indicator of economic size and development in Sweden. Estimates of annual GDP per capita in 1910/1912 constant price were acquired from the Swedish Historical National Accounts (SHNA) (Lobell *et al.*, 2008; Schön and Krantz, 2012). Economic historians have pointed out several pitfalls in the SHNA (Bohlin, 2003; Myrdal and Morell, 2011; Edvinsson, 2013a), therefore we made use of the percentage volume growth in GDP (GDP volume growth) as an alternative measure of economic size (Edvinsson, 2013a,b). We also used consumer price index (CPI) as a measure of changes in price levels of a basket of consumer goods and services purchased by households to better capture the living cost and reflect the level of stress among Swedish people. An estimation of Swedish CPI (index 1914 = 100) was obtained from Edvinsson and Söderberg (2011).

Three economic indicators that are widely employed in historical research were also utilised in this paper. First, we used real wage, the wage adjusted for inflation, as a direct measure of the amount of goods and services that can be bought and a proxy for standard of living in pre-industrial Sweden. We drew on a series of daily real wage of Swedish male labourers from Söderberg (2010). During the pre-industrial period, the predominant part of household consumption was almost always food, and hence the price of food is often treated as another proxy for standard of living. The price of rye, the most important staple food in pre-industrial Sweden, was achieved in Jörberg (1972). Last, CPI, real wage and rye price are determined partly by crop yields which may be used independently as a proxy for population stress (Eckstein *et al.*, 1985). A crop index was computed by Sundbärg (1907) and quantifies the relative abundance of crops in the calendar year season. An index of 3.0 refers to an average crop yields in a specific year, so any year with an index greater/smaller than 3.0 indicates crop yields above/below the average during that year.

In previous research, Catalano and Bruckner (2005) used private consumption in 1985 prices from the Swedish Macro Data from 1861 to 1988 (Institute for International Economic Studies, 2019) and Statistics Sweden (1997) as a measure of the capacity of Swedish households to consume goods and services and a proxy for economic stress. This covariate was retained and labelled as consumption (old) in our analysis. In addition, we drew on updated estimates of consumption from the SHNA and termed this covariate consumption (new) as an alternative measure of consumption to check whether the previous findings are robust.

Following existing research, economic indicators including GDP per capita, CPI, real wage, rye price, consumption (new) and consumption (old) were expressed as annual percentage change (current value relative to the value in last year). We kept the original values of GDP volume growth since it has already been expressed as a percentage change.

### Climatic covariate

To get a picture of abnormal temperature as a population stressor, we relied on temperature anomalies. A temperature anomaly is the difference from an average or baseline temperature. A positive anomaly indicates that the observed temperature is warmer than the baseline, while a negative anomaly indicates the observed temperature was cooler than the baseline, and therefore temperature anomaly can best capture the variation in ambient temperature over a long period. Temperature anomalies (1961–1990 average temperature as baseline) were obtained from the georeferenced CRUTEM4 data set that provides global historical near-surface air temperature anomalies over land (Jones *et al.*, 2012). From the dissemination of CRUTEM4 data via Google Earth (Osborn and Jones, 2014), we extracted time series of annual temperature anomalies over six 5 × 5 grids on which the Swedish territory is located and averaged the six values in a given year to represent the temperature anomaly for the whole of Sweden in that year (Moberg and Alexandersson, 1997). Annual temperature anomalies were computed over 21-month intervals, in other words, the value of temperature anomaly in a year was calculated as the mean of monthly anomalies over 9 months (April to December) in the previous year and over 12 months (January to December) in the current year (Brohan *et al.*, 2006; Helle *et al.*, 2009).

### Control variables

Helle *et al.* (2009) suggested that the TWH should be tested in a multivariate framework so that one can examine the ‘simultaneous role’ of various population stressors. They included not only economic and climatic covariates but also other variables such as mortality in their time-series models. In this article, we also added extra control variables to the baseline models. Importantly, one should be aware that the additional variables included in Helle *et al.* (2009) and in our study are likely to be the outcomes rather than the causes of economic and climatic changes so that these variables are likely to be ‘bad controls’. We treated these multivariate models as additional robustness checks and reported the results in Supplementary data.

The first control variable is annual life expectancy at birth ($e_{0}$) available from the HMD, and in addition to total $e_{0}$, we also used female and male $e_{0}$ separately. $e_{0}$ summaries the overall mortality of the Swedish population in a given year and serves as a proxy for population-level stress with respect to health.

Two other control variables were both gathered from the Human Fertility Collection (Max Planck Institute for Demographic Research and Vienna Institute of Demography, 2021). We used total fertility rate (TFR) as a proxy for birth order and mean age at childbearing (MACB) as a proxy for maternal age, as both have been shown to be negatively correlated with SRB (Chahnazarian, 1988). If there were two or more estimates of TFR or MACB for a given year, we averaged all estimates in that year to get a single measure.

### Statistical analyses

We performed three sets of analyses to examine the correlation between SRB and maternal stress in this paper: Analysis I covers the period 1749–1991; Analysis II covers the period 1749–1861; and Analysis III covers the period 1862–1991. The study period of Analysis I is based on the time span of available SRB data; the study period of Analysis II can be roughly seen as the pre-industrial era of Sweden (Edvinsson, 2017); and the study period of Analysis III is identical to that of Catalano and Bruckner (2005), which enables us to compare our results with theirs. Data availability of covariates varies across the three analyses, and accordingly Analysis I contains the following covariates: GDP per capita, GDP volume growth and CPI; Analysis II contains: GDP per capita, GDP volume growth, CPI, real wage, rye price and crop index; and Analysis III contains: GDP per capita, GDP volume growth, CPI, private consumption (new), private consumption (old) and temperature anomaly.

In the multivariate models as robustness checks, we added control variables to each of the baseline models in Analyses I, II and III. $e_{0}$ was added to all three analyses, and we used total, female and male $e_{0}$ in separate models to avoid multicollinearity problems. TFR and MACB were added only to Analysis III because they are available only from 1850. In addition, data on $e_{0}$ are available only from 1751 so Analyses I and II in robustness checks started from 1751 instead of 1749.

To test the foetal loss mechanism, we replicated Analysis III and replaced the outcome variable by annual sex ratio of stillbirths. Because data on stillbirths are only available from 1861, we are unable to test this mechanism in the pre-industrial era.

For each of the analyses, we employed an ARIMA (autoregressive integrated moving average) approach to modelling the relationship between SRB and each covariate. Given the fact that foetuses conceived after April in one year are likely to be born in the next year, we also included a 1-year lag in covariates in our ARIMA models. We did not apply the lag term to temperature anomaly since its value in the present year has already taken into account the temperatures in the previous year.

All statistical analyses were conducted using R version 3.4.4 (R Core Team, 2018). To perform our analyses, we first tested the stationarity assumption of all variables being modelled using the augmented Dickey–Fuller test, which tests the null hypothesis that a unit root is present in a time series process and therefore the process is non-stationary. Once we confirmed that all covariates are stationary processes, we estimated the ARIMA model with optimal orders of autoregressive, differencing and moving average chosen by the ‘auto.arima()’ function in the R package ‘forecast’ (Hyndman and Khandakar, 2008).

## Results

### Summary statistics

**Figure 1.**
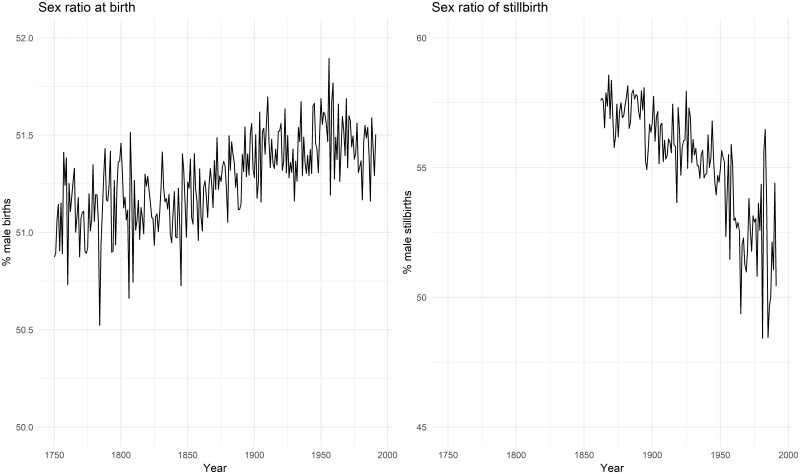
**Trends in sex ratio at birth (1749–1991) and sex ratio of stillbirths in Sweden (1862–1991)**.

**Figure 2.**
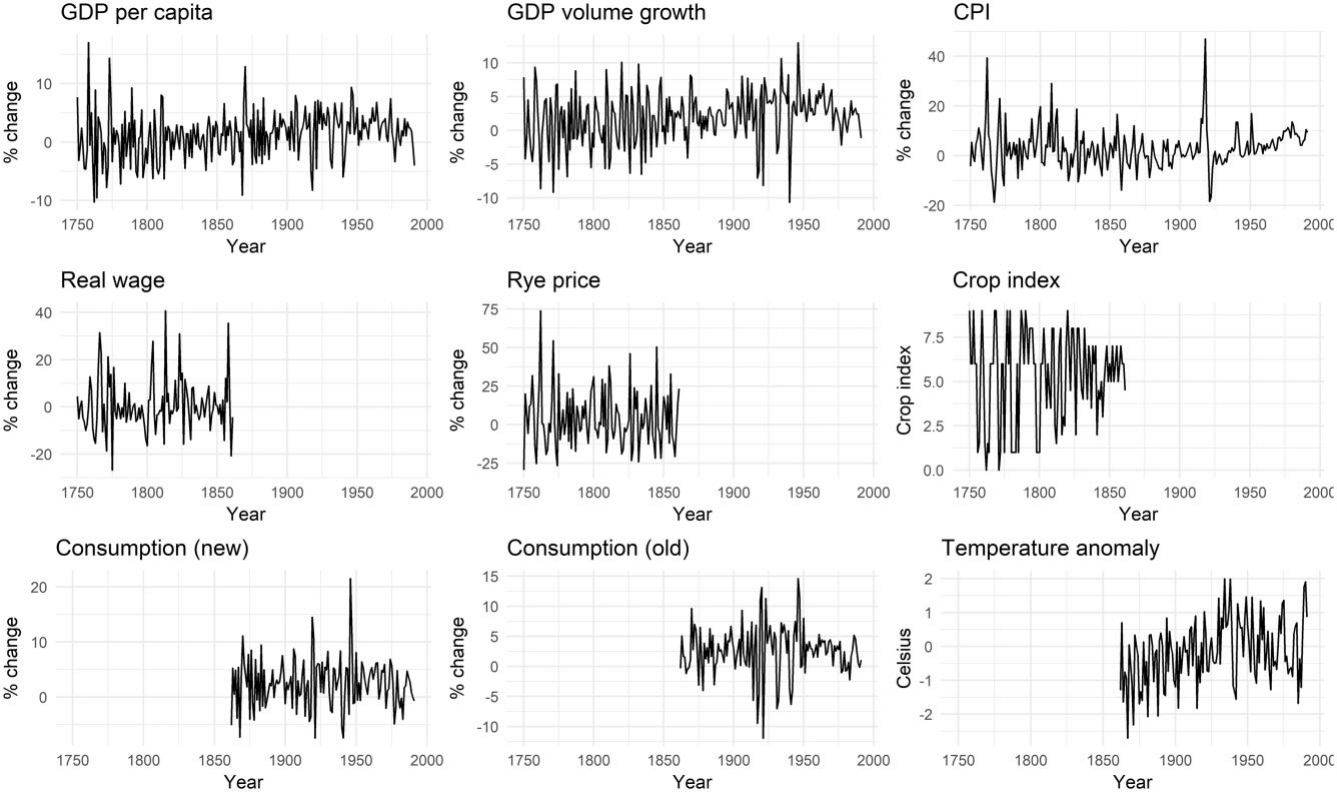
**Trends in economic and climatic covariates in Sweden (1749–1991), (1749–1861) and (1862–1991)**.

Figure 1 depicts temporal changes in Swedish SRB (left) and sex ratio of stillbirths (right). The values of SRB in 1749–1991 vary between 50.5% and 51.9% with a mean of 51.3 and a standard deviation of 0.22. A slightly increasing trend can be detected prior to 1950. The values of sex ratio of stillbirths in 1862–1991 fluctuate between 48.4% and 58.5% with a mean of 55.2 and a standard deviation of 2.25. The variation in sex ratio of stillbirths is appreciably larger than that of SRB, and a descending trend can be observed before 1950. Figure 2 shows changes in nine economic and climatic covariates in Sweden. Table I shows summary statistics of and results from the augmented Dickey–Fuller tests on outcome variables and covariates. By comparing the augmented Dickey–Fuller test statistics with recommended critical value (−3.51 at 1% with a sample size of 100 observations), the null hypothesis is rejected in favour of the alternative hypothesis that all covariates are stationary and thus are suitable for ARIMA model. Correlation matrices of the covariates used in three analyses are shown in Supplementary Tables SI, SII, and SIII; it can be seen that apart from a few covariates, the correlation between most covariates is below 0.7, indicating that these covariates do capture potentially different stressors.

**Table I:** Summary statistics and augmented Dickey–Fuller test statistics of outcome variables and covariates.

	1749–1991			1749–1861			1862–1991		
	Mean	SD	ADF	Mean	SD	ADF	Mean	SD	ADF
**Outcome variables**									
SRB	51.27	0.22	−5.10	51.12	0.18	−5.07	51.40	0.16	−3.46
Sex ratio of stillbirths							55.20	2.25	−3.40
**Covariates**									
GDP per capita	1.29	4.06	−7.11	0.37	4.44	−5.88	2.08	3.53	−5.64
GDP volume growth	1.94	3.85	−7.59	1.06	4.21	−7.69	2.71	3.35	−5.82
CPI	2.89	7.88	−5.48	2.64	8.70	−5.50	3.11	7.11	−4.62
Real wage				0.63	11.19	−6.16			
Rye price				3.77	18.47	−6.84			
Crop index				5.29	2.52	−5.29			
Consumption (new)							2.51	4.45	−5.39
Consumption (old)							2.53	4.05	−6.28
Temperature anomaly							−0.16	0.95	−4.70

ADF, augmented Dickey–Fuller test statistics; CPI, consumer price index; GDP, gross domestic product; SD, standard deviation; SRB, sex ratio at birth.

### Analysis I: 1749–1991

Results from Analysis I are shown in Table II. We can see that none of the coefficients are statistically significant. The coefficients for GDP per capita and GDP volume growth are negative (except that for GDP volume growth with no lag time) while the coefficients for CPI are positive. The directions of these coefficients are opposite to the TWH’s prediction that SRB should be positively associated with GDP and negatively associated with CPI because SRB should decline during an economic contraction when GDP growth decelerates and price level rises.

**Table II:** Coefficients from regression models predicting Swedish sex ratio at birth (calculated as proportion of male births), 1749–1991.

	Outcome variable: SRB, 1749–1991		
GDP per capita, *t*	−0.0016		
	(0.0026)		
GDP per capita, *t*-1	−0.0020		
	(0.0026)		
GDP volume growth, *t*		0.0007	
		(0.0028)	
GDP volume growth, *t*-1		−0.0014	
		(0.0028)	
CPI, *t*			0.0002
			(0.0015)
CPI, *t*-1			0.0024
			(0.0015)

ARIMA (p,d,q)	(1,1,2)	(1,1,2)	(1,1,2)
Ljung-Box Q test	7.51	7.47	8.41
AIC	−166.00	−165.48	−168.30

Standard errors in parentheses.

ARIMA, autoregressive integrated moving average; AIC, Akaike information criterion; CPI, consumer price index; GDP, gross domestic product; SRB, sex ratio at birth; *t*, no lag in time between covariates; *t*-1, 1-year lag between covariates.

### Analysis II: 1749–1861

Results from Analysis II are shown in Table III. Again, most coefficients are not significant, and the signs of coefficients for GDP per capita, GDP volume growth, CPI and crop index (at a 1-year lag) are in the opposite direction compared to that suggested by the TWH. Real wage and rye price at a 1-year lag 1 are significant at 10% and 5%, respectively (*P *=* *0.058 and 0.032). A 1% increase in real wage is associated with a 0.003% reduction in the proportion of male births in the following year, while 1% increase in rye price is associated with a 0.002% increase in the proportion of male births in the subsequent year. These results are inconsistent with the TWH, according to which economic improvements, as proxies for reduced maternal stress, should be linked with increases in SRB.

**Table III:** Coefficients from regression models predicting Swedish sex ratio at birth (calculated as proportion of male births), 1749–1861.

	Outcome variable: SRB, 1749–1861					
GDP per capita, *t*	−0.0063					
	(0.0039)					
GDP per capita, *t*-1	−0.0027					
	(0.0039)					
GDP volume growth, *t*		−0.0017				
		(0.0041)				
GDP volume growth, *t*-1		−0.0031				
		(0.0041)				
CPI, *t*			0.0010			
			(0.0020)			
CPI, *t*-1			0.0031			
			(0.0020)			
Real wage, *t*				−0.0016		
				(0.0015)		
Real wage, *t*-1				−0.0029^†^		
				(0.0015)		
Rye price, *t*					−0.0001	
					(0.0009)	
Rye price, *t*-1					0.0020^*^	
					(0.0009)	
Crop index, *t*						0.0016
						(0.0074)
Crop index, *t*-1						−0.0042
						(0.0073)

ARIMA (p,d,q)	(0,0,0)	(0,0,0)	(0,0,0)	(0,0,0)	(0,0,0)	(0,0,0)
Ljung-Box Q test	5.55	5.38	6.44	4.48	6.67	5.78
AIC	−56.78	−54.74	−57.17	−58.96	−58.58	−54.36

Standard errors in parentheses.

† p<0.1 ;

* p<0.05 .

ARIMA, autoregressive integrated moving average; CPI, consumer price index; GDP, gross domestic product; SRB, sex ratio at birth; *t*, no lag in time between covariates; *t*-1, 1-year lag between covariates.

### Analysis III: 1862–1991

Results from Analysis III are shown in Table IV. The only significant covariate is consumption (old) (*P *=* *0.004), the covariate used in Catalano and Bruckner (2005). A 1% increase in percentage change in private consumption is associated with a 0.008% increase in SRB in the current year, which is qualitatively identical to the findings from Catalano and Bruckner (2005) and in line with the TWH. It should also be noted that consumption (new), the alternative and updated measure of consumption, shows no statistically significant association with SRB, despite its high correlation with the old measure (*r *=* *0.79, *P *<* *0.001). Temperature anomaly is positively correlated with SRB, indicating that more sons should be born in warmer years, but we need to interpret this with extra caution as the coefficient is not statistically significant.

**Table IV:** Coefficients from regression models predicting Swedish sex ratio at birth (calculated as proportion of male births), 1862–1991.

	Outcome variable: SRB, 1862–1991					
GDP per capita, *t*	0.0044					
	(0.0035)					
GDP per capita, *t*-1	−0.0018					
	(0.0035)					
GDP volume growth, *t*		0.0046				
		(0.0036)				
GDP volume growth, *t*-1		−0.0003				
		(0.0037)				
CPI, *t*			−0.0012			
			(0.0022)			
CPI, *t*-1			0.0019			
			(0.0022)			
Consumption (new), *t*				0.0032		
				(0.0028)		
Consumption (new), *t*-1				−0.0006		
				(0.0028)		
Consumption (old), *t*					0.0083^*^	
					(0.0029)	
Consumption (old), *t*-1					−0.0021	
					(0.0029)	
Temperature anomaly, *t*						0.0153
						(0.0136)

ARIMA (p,d,q)	(0,1,1)	(1,1,1)	(1,1,1)	(0,1,1)	(1,0,2)	(1,1,1)
Ljung-Box Q test	9.15	7.33	8.79	9.13	6.76	8.06
AIC	−124.43	−124.16	−123.28	−124.03	−127.59	−125.78

Standard errors in parentheses.

* P<0.01 .

ARIMA, autoregressive integrated moving average; CPI, consumer price index; GDP, gross domestic product; SRB, sex ratio at birth; *t*, no lag in time between covariates; *t*-1, 1-year lag between covariates.

### Multivariate models as robustness checks

Trends in total, female and male $e_{0}$ as well as TFR and MACB are illustrated in Supplementary Fig. SI, from which we can visibly see upward trends in $e_{0}$ and downward trends in TFR and MACB, showing the demographic transition in Sweden. Clearly, all of the control variables are not stationary so we had to difference them first. The number of differences required to make these time series stationary was selected through the ‘ndiffs()’ function in the R package ‘forecast’. MACB required a second-order differencing, while all others needed to be differenced just once.

Results from multivariate models for the period 1752–1991 controlling for total, female and male $e_{0}$ are shown in Supplementary Tables SIV, V, and VI, respectively. We can see that all of the three economic covariates still show no significant correlation with SRB. Life expectancies are themselves not significant.

Results from multivariate models for the period 1752–1861 are presented in Supplementary Tables SVII, SVIII, and SIX. Again, life expectancies are not significant, and the only significant (at 10%) covariate is rye price at a 1-year lag, while the sign of its coefficient is inconsistent with the TWH’s prediction.

Results from robustness checks on Analysis III (1862–1991) controlling for life expectancies, TFR and MACB are reported in Supplementary Tables SX, SXI, and SXII. In all but one case, MACB as a proxy for maternal age is negatively and significantly correlated with SRB between 1862 and 1991, while all other control variables are not. The only exception where MACB is not statistically significant is the models using the old measure of private consumption, the same economic proxy used in Catalano and Bruckner (2005). Similar to Analysis III, consumption (old) is the only variable that is significantly associated with SRB, and all other economic and climatic proxies show no significant association with SRB.

### Foetal loss mechanism

Table V presents results from analysis of the correlation between sex ratio of stillbirths and proxies for maternal stress for the period 1862–1991. None of the coefficients are statistically significant, suggesting no evidence that maternal stress reduces the SRB by increasing male foetal loss.

**Table V:** Coefficients from regression models predicting Swedish sex ratio of stillbirths (calculated as proportion of male stillbirths), 1862–1991.

	Outcome variable: sex ratio of stillbirths, 1862–1991					
GDP per capita, *t*	0.0035					
	(0.0312)					
GDP per capita, *t*-1	−0.0191					
	(0.0313)					
GDP volume growth, *t*		0.0051				
		(0.0332)				
GDP volume growth, *t*-1		−0.0516				
		(0.0333)				
CPI, *t*			−0.0137			
			(0.0200)			
CPI, *t*-1			0.0165			
			(0.0200)			
Consumption (new), *t*				−0.0181		
				(0.0247)		
Consumption (new), *t*-1				−0.0260		
				(0.0247)		
Consumption (old), *t*					0.0013	
					(0.0272)	
Consumption (old), *t*-1					−0.0271	
					(0.0272)	
Temperature anomaly, *t*						0.1813
						(0.1265)

ARIMA (p,d,q)	(0,1,1)	(0,1,1)	(0,1,1)	(0,1,1)	(0,1,1)	(0,1,1)
Ljung-Box Q test	12.69	14.78	11.75	13.48	13.51	12.55
AIC	444.70	442.70	444.32	443.57	441.72	441.03

Standard errors in parentheses.

ARIMA, autoregressive integrated moving average; CPI, consumer price index; GDP, gross domestic product; *t*, no lag in time between covariates; *t*-1, 1-year lag between covariates.

## Discussion

This study uses time series models to analyse the relationship between Swedish SRB and a number of economic and climatic proxies for maternal stress over 243 years between 1749 and 1991. The vast majority of covariates are not statistically significant in any of the three study periods, 1749–1991, 1749–1861 and 1862–1991, indicating that the relationship between maternal stress and SRB is extremely weak or even non-existing. Only three covariates are statistically significant: real wage and rye price in 1749–1861 and consumption (old) in 1862–1991. However, real wage and rye price are just significant at 10% and 5%, respectively, and the signs of the coefficients conflict with the prediction of the TWH, leaving consumption (old) as the only significant covariate in support of the TWH. Additional analysis finds no significant relationship between the sex ratio of stillbirths and maternal stress in 1862–1991.

Long-term time series data on SRB are available in several Nordic countries, but the previous research is either entirely descriptive (e.g. only looking at the temporal trends and fluctuations in SRB without any explanatory variables) or limited to a specific group of the population that may not be representative (Vartiainen *et al.*, 1999; Helle *et al.*, 2007; Fellman and Eriksson, 2011). This article is the first to perform an exploratory analysis of SRB change and associated factors as well as the first to test the TWH over such a long duration. Analysis I examines the longest continuous time series of SRB from 1749 to 1991 and its association with three economic indicators as proxies for population stress. Our results suggest no evidence for the TWH as all of the coefficients in Analysis I are not significant. The results threaten the external validity of many existing studies with a much shorter period under investigation since the supportive evidence for the TWH reported in those articles may only hold within their study periods and cannot be generalised to other times (e.g. a pre-industrial era).

A large body of literature has identified substantial demographic responses, such as fertility, mortality and marriage, to economic variations such as wages and prices in the pre-industrial period (Bengtsson and Ohlsson, 1985; Eckstein *et al.*, 1985; Galloway, 1988; Larsen, 1988; Edvinsson, 2017). The estimated relationship between demographic rates and economic conditions is usually more pronounced during the pre-industrial era than during industrialisation or the post-industrial time. Analysis II contributes to this vein of research by adding a key demographic outcome that has often been overlooked in this historical period, SRB. Unlike other demographic variables, we did not find evidence that trends in SRB respond to changes in a number of economic indicators such as GDP, CPI and crop yields. The results are surprising since in the absence of the welfare state and economic development that help women cope with economic stress, we would expect SRB to be more responsive to economic fluctuations in the pre-industrial period, based on the TWH. Although two economic variables, real wage and rye price, showed a statistically significant association with SRB, the coefficients were in the opposite direction compared to the TWH’s prediction.

The study period of Analysis III is 1862–1991, exactly the same as the study period of Catalano and Bruckner (2005). While they used only a single covariate, this study uses six variables covering different dimensions of maternal stress. Catalano and Bruckner (2005) show that Swedish SRB is positively associated with the old measure of consumption with both no lag time and at a 1-year lag. This study agrees on their results when only considering consumption at no lag time, whereas all five other covariates, including an updated and preferred measure of consumption, are not significantly correlated with SRB, indicating that the results from Catalano and Bruckner (2005) are not robust to alternative measures of maternal stress. Given that almost all earlier research on the TWH relies on a single measure of maternal stress, their results are very likely to be speculative and require further robustness checks.

Analysis III also adds to the empirical literature on the relationship between SRB and temperature. In line with previous research (Helle *et al.*, 2007; Catalano *et al.*, 2008), we also found that warmer years are associated with more male births, but this association was not significant. One possible reason for the disagreement between the results regarding statistical significance is that compared with the indicators used in previous research, we rely on a more precise indicator, temperature anomaly, that covers broader areas (i.e. the entire Swedish territory) and better reflects the climatic shock to the population caused by abnormal temperature that deviates from time trends.

In the multivariate models, most of the explanatory variables, including the control variables, still show no significant association with SRB, indicating that our findings are robust to alternative specifications controlling for a number of confounders that may potentially affect SRB. Interestingly, female life expectancy at birth as a proxy for maternal stress resulting from mortality and health conditions also shows no significant correlation with SRB, providing further evidence against the TWH. According to the aforementioned demographic literature (e.g. Galloway, 1988), demographic rates and variables are often responsive to socioeconomic and climatic changes, so changes in the economic and climatic covariates would possibly cause changes in the control variables. Hence, these demographic variables are probably ‘bad controls’ and the results from these multivariate models should be interpreted with caution.

Earlier literature has proposed that maternal stress reduces SRB through increasing male foetal loss (James and Grech, 2017), which has been verified using data on foetal deaths from the United States (Catalano *et al.*, 2005a; Bruckner *et al.*, 2010). While the two American studies only cover a study period of about a decade, we examine the relationship between stillbirth sex ratio and various proxies for maternal stress in Sweden over a century. We do not find a significant correlation between them, again questioning the external validity of existing research.

In addition to the impact of maternal stress on human SRB, one may also consider the influence of the father whose sperm contributes either an X chromosome that leads to a male offspring (XX-paired chromosomes) or a Y chromosome that leads to a female offspring (XY-paired chromosomes). The features of male-provided sperm are important in determining the sex ratio at conception, or the primary sex ratio (PSR), and paternal stress may affect sperm and then alter PSR. Unfortunately, there is a lack of data on sperm quality and PSR in Sweden, so we cannot formally test this mechanism, however, we still believe that there is no evidence of the effect of paternal stress on offspring sex ratio for three reasons. First, it has generally been thought that X- and Y-bearing sperm are produced in equal number and have an equal possibility of insemination, and hence the PSR should be 0.5 (James, 2012). Using detailed data from a variety of sources, a comprehensive analysis of the trajectory of the human sex ratio from conception to birth also reveals that the PSR is unbiased in terms of sex (Orzack *et al.*, 2015). Second, the economic and climatic covariates used in this study are at the national level so that they affect women and men simultaneously and can be seen as proxies for both maternal and paternal stress. Furthermore, the real wage modelled in Analysis II is based on the daily real wage of Swedish male labourers so that it is related directly to men. Given the lack of statistical significance in our findings, we think there is no evidence of the father’s aspect of the TWH. Last, in the robustness checks, we show that male life expectancy at birth, a measure of male mortality and health conditions, is not significantly associated with SRB, indicating no evidence that the level of paternal stress resulting from poor health conditions is linked with trends in SRB.

This study encounters a few limitations and concerns about data quality. First, annual data are used in the analysis, and we cannot investigate the potential seasonality in SRB due to the lack of disaggregated monthly data. Another limitation is the ecological fallacy. This study uses national data and provides macro-level evidence that may not necessarily be true for all individuals. A related study using individual-level data from Swedish administrative registers available for a shorter duration from 1960 onward found no statistically significant association between offspring sex and maternal socioeconomic status (Kolk and Schnettler, 2016). A further investigation into historical period in this topic can be conducted given the enormous individual data available for pre-industrial Sweden, and the proxy for maternal stress can be socioeconomic status like occupations, at the individual level, or local food price, at the aggregate level. Last, the parents’ preference for the offspring’s sex may lead to sex-selective infanticide and abortion and thus cause inaccuracy in SRB records. Nevertheless, the literature has suggested that infanticide has been rare in Sweden since the early 18th century (Sandin, 2013), and prenatal sex selection is very unlikely in contemporary Sweden because of a small or non-existent son preference (Miranda *et al.*, 2018) and the government’s critical stance towards sex-selective abortion (Purewal and Eklund, 2018). As a result, it is reasonable to believe that Swedish SRB data are not subject to serious measurement errors due to sex-selective misreporting. Another limitation is that given the social and political stability of Sweden, our findings may not be generalised to the contexts of extreme maternal stress conditions such as famine and war.

## Data availability

The data underlying this article will be shared on reasonable request to the corresponding author.

## Supplementary Material

deab158_Supplementary_Table_S1

deab158_Supplementary_Table_S2

deab158_Supplementary_Table_S3

deab158_Supplementary_Table_S4

deab158_Supplementary_Table_S5

deab158_Supplementary_Table_S6

deab158_Supplementary_Table_S7

deab158_Supplementary_Table_S8

deab158_Supplementary_Table_S9

deab158_Supplementary_Table_S10

deab158_Supplementary_Table_S11

deab158_Supplementary_Table_S12

deab158_Supplementary_Figure_S1
