# Supplementary material for: Maternal stress and sex ratio at birth in Sweden over two and a half centuries: a retest of the Trivers–Willard hypothesis
Source: Hum Reprod. 2021 Jul 26;36(10):2782–92. doi: 10.1093/humrep/deab158 (PMC8648295; doi:10.1093/humrep/deab158)
Supplement: deab158_Supplementary_Table_S1 [file deab158_supplementary_table_s1.pdf]

Human Reproduction, pp. 1–1, 2021
doi:10.1093/humrep/deab158

human reproduction

SUPPLEMENTARY DATA

Supplementary Table SI Correlation matrix of covariates used in Analysis I, 1749–1991.

	GDP per capita	GDP volume growth	CPI
GDP per capita	1.00		
GDP volume growth	0.58***	1.00	
CPI	−0.30***	−0.46***	1.00

***$P < 0.001$.
CPI, consumer price index; GDP, gross domestic product.
