# Supplementary material for: Maternal stress and sex ratio at birth in Sweden over two and a half centuries: a retest of the Trivers–Willard hypothesis
Source: Hum Reprod. 2021 Jul 26;36(10):2782–92. doi: 10.1093/humrep/deab158 (PMC8648295; doi:10.1093/humrep/deab158)
Supplement: deab158_Supplementary_Table_S2 [file deab158_supplementary_table_s2.pdf]

Human Reproduction, pp. 1–1, 2021
doi:10.1093/humrep/deab158

human reproduction

SUPPLEMENTARY DATA

Supplementary Table SII Correlation matrix of covariates used in Analysis II, 1749–1861.

	GDP per capita	GDP volume growth	CPI	Real wage	Rye price	Crop index
GDP per capita	1.00					
GDP volume growth	0.43***	1.00				
CPI	−0.37***	−0.63***	1.00			
Real wage	0.23*	0.48***	−0.65***	1.00		
Rye price	−0.39***	−0.77***	0.88***	−0.63***	1.00	
Crop index	0.26**	0.63***	−0.56***	0.38***	−0.60***	1.00

*$P < 0{:}05$; **$P < 0{:}01$; ***$P < 0.001$.
CPI, consumer price index; GDP, gross domestic product.
