# Supplementary material for: Maternal stress and sex ratio at birth in Sweden over two and a half centuries: a retest of the Trivers–Willard hypothesis
Source: Hum Reprod. 2021 Jul 26;36(10):2782–92. doi: 10.1093/humrep/deab158 (PMC8648295; doi:10.1093/humrep/deab158)
Supplement: deab158_Supplementary_Table_S3 [file deab158_supplementary_table_s3.pdf]

Human Reproduction, pp. 1–1, 2021
doi:10.1093/humrep/deab158

human reproduction

SUPPLEMENTARY DATA

Supplementary Table SIII Correlation matrix of covariates used in Analysis III, 1862–1991.

	GDP per capita	GDP volume growth	CPI	Consumption (new)	Consumption (old)	Temperature anomaly
GDP per capita	1.00					
GDP volume growth	0.75***	1.00				
CPI	−0.24**	−0.28**	1.00			
Consumption (new)	0.81***	0.65***	−0.14	1.00		
Consumption (old)	0.69***	0.75***	−0.14	0.79***	1.00	
Temperature anomaly	0.15†	0.28**	−0.06	0.06	0.15†	1.00

†$P < 0{:}1$; **$P < 0.01$; ***$P < 0.001$.
CPI, consumer price index; GDP, gross domestic product.
