# Supplementary material for: Maternal stress and sex ratio at birth in Sweden over two and a half centuries: a retest of the Trivers–Willard hypothesis
Source: Hum Reprod. 2021 Jul 26;36(10):2782–92. doi: 10.1093/humrep/deab158 (PMC8648295; doi:10.1093/humrep/deab158)
Supplement: deab158_Supplementary_Table_S5 [file deab158_supplementary_table_s5.pdf]

Human Reproduction, pp. 1–1, 2021
doi:10.1093/humrep/deab158

human reproduction

SUPPLEMENTARY DATA

Supplementary Table SV Robustness checks controlling for female life expectancy at birth: coefficients from regression models predicting Swedish sex ratio at birth (calculated as proportion of male births), 1752–1991.

		Outcome variable: SRB, 1752–1991	
GDP per capita, *t*	−0.0005		
	(0.0052)		
GDP per capita, *t*-1	0.0001		
	(0.0052)		
GDP volume growth, *t*		0.0048	
		(0.0054)	
GDP volume growth, *t*-1		−0.0036	
		(0.0054)	
CPI, *t*			0.0017
			(0.0026)
CPI, *t*-1			−0.0014
			(0.0027)
Female life expectancy	0.0044	0.0041	0.0080
	(0.0075)	(0.0073)	(0.0065)
ARIMA (p,d,q)	(1,0,1)	(1,0,1)	(1,0,1)
Ljung-Box Q test	8.46	8.63	10.04
AIC	148.34	147.44	148.54

Standard errors in parentheses. ARIMA, autoregressive integrated moving average; CPI, consumer price index; GDP, gross domestic product; SRB, sex ratio at birth; *t*, no lag in time between covariates; *t*-1, 1-year lag between covariates.
