# Supplementary material for: Maternal stress and sex ratio at birth in Sweden over two and a half centuries: a retest of the Trivers–Willard hypothesis
Source: Hum Reprod. 2021 Jul 26;36(10):2782–92. doi: 10.1093/humrep/deab158 (PMC8648295; doi:10.1093/humrep/deab158)
Supplement: deab158_Supplementary_Table_S6 [file deab158_supplementary_table_s6.pdf]

**Human Reproduction, pp. 1–1, 2021**
doi:10.1093/humrep/deab158

human reproduction

## SUPPLEMENTARY DATA

**Supplementary Table SVI Robustness checks controlling for male life expectancy at birth: coefficients from regression models predicting Swedish sex ratio at birth (calculated as proportion of male births), 1752–1991.**

| | Outcome variable: SRB, 1752–1991 | | |
|---|---|---|---|
| GDP per capita, *t* | −0.0005 | | |
| | (0.0052) | | |
| GDP per capita, *t*-1 | −0.0001 | | |
| | (0.0052) | | |
| GDP volume growth, *t* | | 0.0037 | |
| | | (0.0055) | |
| GDP volume growth, *t*-1 | | −0.0042 | |
| | | (0.0054) | |
| CPI, *t* | | | 0.0019 |
| | | | (0.0026) |
| CPI, *t*-1 | | | −0.0016 |
| | | | (0.0027) |
| Male life expectancy | 0.0077 | 0.0070 | 0.0100 |
| | (0.0078) | (0.0074) | (0.0068) |
| ARIMA (p,d,q) | (1,0,2) | (1,0,1) | (1,0,1) |
| Ljung-Box Q test | 8.74 | 9.01 | 10.50 |
| AIC | 149.58 | 147.24 | 147.95 |

Standard errors in parentheses. ARIMA, autoregressive integrated moving average; CPI, consumer price index; GDP, gross domestic product; SRB, sex ratio at birth; *t*, no lag in time between covariates; *t*-1, 1-year lag between covariates.
