# Supplementary material for: Maternal stress and sex ratio at birth in Sweden over two and a half centuries: a retest of the Trivers–Willard hypothesis
Source: Hum Reprod. 2021 Jul 26;36(10):2782–92. doi: 10.1093/humrep/deab158 (PMC8648295; doi:10.1093/humrep/deab158)
Supplement: deab158_Supplementary_Table_S7 [file deab158_supplementary_table_s7.pdf]

Human Reproduction, pp. 1–1, 2021
doi:10.1093/humrep/deab158

human reproduction

SUPPLEMENTARY DATA

Supplementary Table SVII Robustness checks controlling for total life expectancy at birth: coefficients from regression models predicting Swedish sex ratio at birth (calculated as proportion of male births), 1752–1861.

	Outcome variable: SRB, 1752–1861					
GDP per capita, *t*	−0.0060					
	(0.0079)					
GDP per capita, *t*-1	0.0093					
	(0.0083)					
GDP volume growth, *t*		−0.0077				
		(0.0086)				
GDP volume growth, *t*-1		−0.0081				
		(0.0090)				
CPI, *t*			0.0043			
			(0.0039)			
CPI, *t*-1			0.0006			
			(0.0038)			
Real wage, *t*				−0.0047		
				(0.0031)		
Real wage, *t*-1				0.0016		
				(0.0031)		
Rye price, *t*					0.0017	
					(0.0018)	
Rye price, *t*-1					0.0033†	
					(0.0019)	
Crop index, *t*						−0.0076
						(0.0132)
Crop index, *t*-1						0.0075
						(0.0131)
Total life expectancy	0.0029	0.0047	0.0027	0.0093	0.0076	−0.0021
	(0.0104)	(0.0096)	(0.0091)	(0.0092)	(0.0092)	(0.0089)
ARIMA (p,d,q)	(0,0,1)	(3,0,0)	(3,0,0)	(0,0,1)	(3,0,0)	(3,0,0)
Ljung-Box Q test	7.05	15.05	12.10	8.83	18.22	10.84
AIC	95.85	114.81	114.41	92.88	110.26	117.42

Standard errors in parentheses. $^{\dagger}P < 0.1$; ARIMA, autoregressive integrated moving average; CPI, consumer price index; GDP, gross domestic product; SRB, sex ratio at birth; *t*, no lag in time between covariates; *t*-1, 1-year lag between covariates.
