# Supplementary material for: Maternal stress and sex ratio at birth in Sweden over two and a half centuries: a retest of the Trivers–Willard hypothesis
Source: Hum Reprod. 2021 Jul 26;36(10):2782–92. doi: 10.1093/humrep/deab158 (PMC8648295; doi:10.1093/humrep/deab158)
Supplement: deab158_Supplementary_Table_S8 [file deab158_supplementary_table_s8.pdf]

Human Reproduction, pp. 1–1, 2021
doi:10.1093/humrep/deab158

human reproduction

SUPPLEMENTARY DATA

Supplementary Table SVIII Robustness checks controlling for female life expectancy at birth: coefficients from regression models predicting Swedish sex ratio at birth (calculated as proportion of male births), 1752–1861.

	Outcome variable: SRB, 1752–1861					
GDP per capita, *t*	−0.0124					
	(0.0085)					
GDP per capita, *t*-1	0.0046					
	(0.0090)					
GDP volume growth, *t*		−0.0076				
		(0.0086)				
GDP volume growth, *t*-1		−0.0080				
		(0.0090)				
CPI, *t*			0.0043			
			(0.0039)			
CPI, *t*-1			0.0006			
			(0.0038)			
Real wage, *t*				−0.0048		
				(0.0031)		
Real wage, *t*-1				0.0016		
				(0.0031)		
Rye price, *t*					0.0017	
					(0.0018)	
Rye price, *t*-1					0.0033†	
					(0.0019)	
Crop index, *t*						−0.0076
						(0.0132)
Crop index, *t*-1						0.0075
						(0.0131)
Female life expectancy	0.0000	0.0045	0.0026	0.0099	0.0075	−0.0023
	(0.0100)	(0.0095)	(0.0090)	(0.0092)	(0.0091)	(0.0088)
ARIMA (p,d,q)	(3,0,0)	(3,0,0)	(3,0,0)	(0,0,1)	(3,0,0)	(3,0,0)
Ljung-Box Q test	10.82	14.91	12.01	8.88	18.05	10.85
AIC	115.64	114.83	114.42	92.76	110.26	117.42

Standard errors in parentheses. $^{\dagger}P < 0.1$; ARIMA, autoregressive integrated moving average; CPI, consumer price index; GDP, gross domestic product; SRB, sex ratio at birth; *t*, no lag in time between covariates; *t*-1, 1-year lag between covariates.
