# Supplementary material for: Maternal stress and sex ratio at birth in Sweden over two and a half centuries: a retest of the Trivers–Willard hypothesis
Source: Hum Reprod. 2021 Jul 26;36(10):2782–92. doi: 10.1093/humrep/deab158 (PMC8648295; doi:10.1093/humrep/deab158)
Supplement: deab158_Supplementary_Table_S10 [file deab158_supplementary_table_s10.pdf]

Human Reproduction, pp. 1–1, 2021
doi:10.1093/humrep/deab158

human reproduction

SUPPLEMENTARY DATA

Supplementary Table SX Robustness checks controlling for total life expectancy at birth, total fertility rate and mean age at childbearing: coefficients from regression models predicting Swedish sex ratio at birth (calculated as proportion of male births), 1862–1991.

	Outcome variable: SRB, 1862–1991					
GDP per capita, *t*	0.0020					
	(0.0037)					
GDP per capita, *t*-1	−0.0033					
	(0.0035)					
GDP volume growth, *t*		0.0036				
		(0.0036)				
GDP volume growth, *t*-1		−0.0024				
		(0.0037)				
CPI, *t*			−0.0016			
			(0.0023)			
CPI, *t*-1			0.0019			
			(0.0021)			
Consumption (new), *t*				0.0008		
				(0.0030)		
Consumption (new), *t*-1				−0.0017		
				(0.0028)		
Consumption (old), *t*					0.0063*	
					(0.0031)	
Consumption (old), *t*-1					−0.0022	
					(0.0029)	
Temperature anomaly, *t*						0.0161
						(0.0134)
Total life expectancy	0.0030	0.0029	0.0021	0.0032	0.0023	0.0047
	(0.0081)	(0.0079)	(0.0086)	(0.0081)	(0.0081)	(0.0079)
TFR	0.0134	0.0209	0.0146	0.0250	0.0034	0.0090
	(0.1095)	(0.1080)	(0.1065)	(0.1099)	(0.1093)	(0.1059)
MACB	−0.3176*	−0.3209*	−0.3293*	−0.3101*	−0.2345	−0.3142*
	(0.1415)	(0.1428)	(0.1432)	(0.1441)	(0.1454)	(0.1391)
ARIMA (p,d,q)	(0,1,1)	(1,1,1)	(1,1,1)	(0,1,1)	(2,0,1)	(1,1,1)
Ljung-Box Q test	7.95	5.82	6.19	8.21	7.70	5.67
AIC	−124.06	−123.98	−123.57	−123.28	−124.62	−126.20

Standard errors in parentheses. *$P < 0.05$; ARIMA, autoregressive integrated moving average; CPI, consumer price index; GDP, gross domestic product; MACB, mean age at childbearing; SRB, sex ratio at birth; *t*, no lag in time between covariates; *t*-1, 1-year lag between covariates; TFR, total fertility rate.
