# Supplementary figures and images for: Maternal stress and sex ratio at birth in Sweden over two and a half centuries: a retest of the Trivers–Willard hypothesis
Source: Hum Reprod. 2021 Jul 26;36(10):2782–92. doi: 10.1093/humrep/deab158 (PMC8648295; doi:10.1093/humrep/deab158)

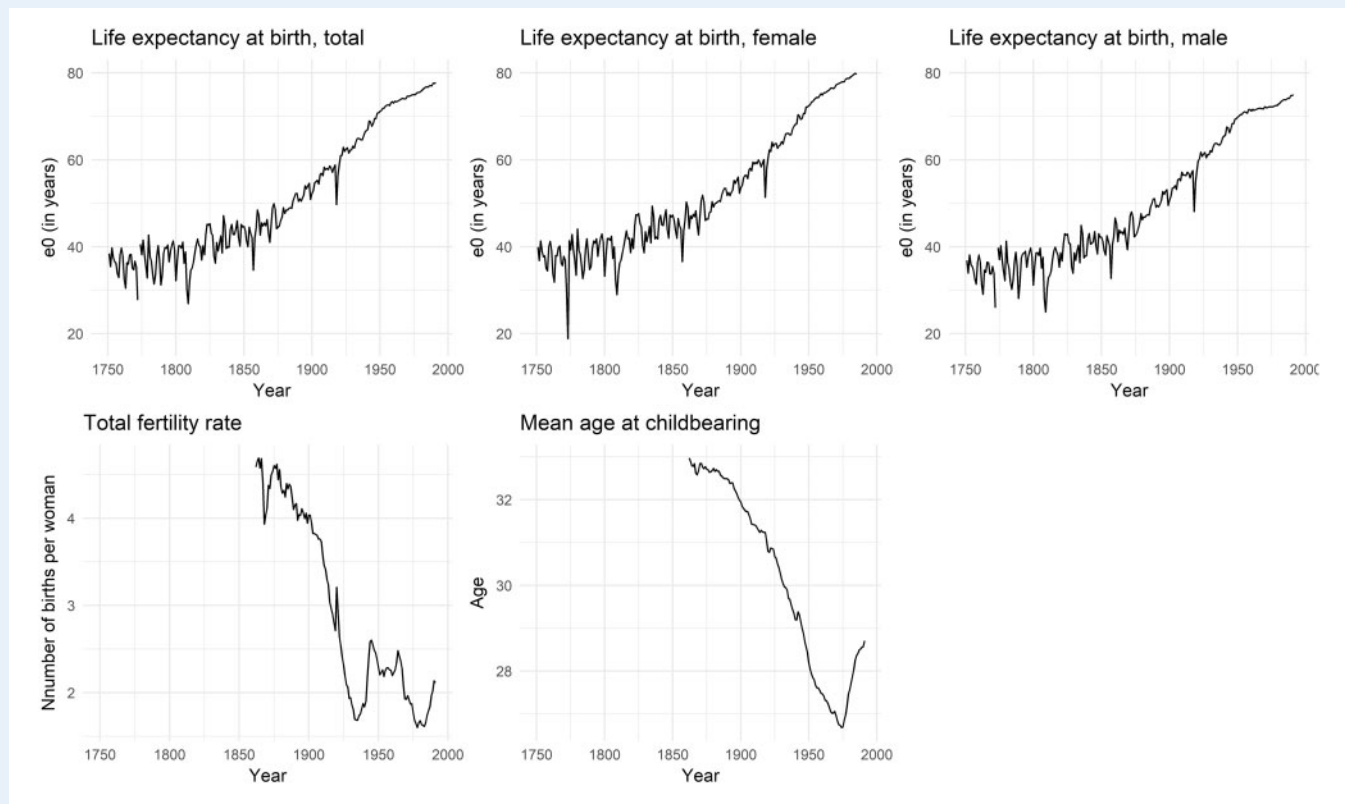

**Supplementary Figure SI.** Trends in control variables in Sweden (1749–1991) and (1862–1991).

Supplement: deab158_Supplementary_Figure_S1 [file deab158_supplementary_figure_s1.pdf]
